# Asymmetric cortical development and prognosis in fetuses with isolated mild fetal ventriculomegaly: an observational prospective study

**DOI:** 10.1186/s12884-021-03692-x

**Published:** 2021-03-10

**Authors:** Rong Zhu, Jun Ya Chen, Xin Lin Hou, Li Li Liu, Guo Yu Sun

**Affiliations:** 1 Department of Obstetrics & Gynecology, Peking University First Hospital, No. 1 Xi-An-Men Street, Xi-Cheng District, Beijing, 100034 China; 2 Department of Pediatrics, Peking University First Hospital, No. 1 Xi-An-Men Street, Xi-Cheng District, Beijing, 100034 China

**Keywords:** Isolated mild fetal ventriculomegaly, Asymmetric prenatal cortical maturation, ‘Catch-up growth’ pattern, Neurodevelopment evaluation, Prognosis

## Abstract

**Background:**

Assessments of cortical development and identifying factors that may result in a poor prognosis for fetuses with isolated mild ventriculomegaly (IMVM) is a hot research topic. We aimed to perform a constant, detailed assessment of cortical development in IMVM fetuses using ultrasound and determine whether asymmetric cortical development occurred. Moreover, we aimed to estimate the prognosis of IMVM fetuses and compare the difference in the prognosis of IMVM fetuses presenting symmetric and asymmetric cortical maturation.

**Methods:**

IMVM was diagnosed by regular ultrasound, neurosonography and fetal MRI. Genetic and TORCH examinations were conducted to exclude common genetic abnormalities and TORCH infection of fetuses. Ultrasound examinations were conducted at an interval of 2–3 weeks to record sulcus development in IMVM fetuses using a scoring system. The neonatal behavioral neurological assessment (NBNA), the Ages and Stages Questionnaire, Third Edition (ASQ-3) and the Bayley Scales of Infant Development, First Edition (BSID-I) were performed after birth.

**Results:**

Forty fetuses with IMVM were included: twenty showed asymmetric cortical maturation and twenty showed symmetric cortical maturation. For IMVM fetuses presenting asymmetric cortical maturation, the mean gestational age (GA) at the first diagnosis of relatively delayed development was 24.23 weeks for the parieto-occipital sulcus, 24.71 weeks for the calcarine sulcus, and 26.43 weeks for the cingulate sulcus. All the sulci with delayed development underwent ‘catch-up growth’ and developed to the same grade as the sulci of the other hemisphere. The mean GA at which the two sides developed to the same grade was 29.40 weeks for the parieto-occipital sulcus, 29.30 weeks for the calcarine sulcus and 31.27 weeks for the cingulate sulcus. The NBNA, ASQ-3 and BSID-I scores of all patients were in the normal range.

**Conclusions:**

IMVM fetuses may show mild asymmetric cortical maturation in the second trimester, but the relatively delayed sulci undergo ‘catch-up growth’. The neurodevelopment of IMVM fetuses presenting asymmetric cortical maturation and ‘catch-up growth’ is not statistically significantly different from IMVM fetuses presenting symmetric cortical maturation.

## Background

Isolated mild ventriculomegaly (IMVM) is defined as mild ventriculomegaly (ventricular atrial width of 10–14.9 mm, regardless of gestational age) in the absence of additional structural abnormalities or chromosomal aberrance [1–4]. According to a recent meta-analysis, the incidence of abnormal or delayed neurodevelopment in infants with IMVM is approximately 7.9% [5]. However, a widely approved method to predict abnormal or delayed postnatal neurodevelopment is not available. The uncertain prognosis of IMVM fetuses leads to difficulties in prenatal consultation, and identifying factors that may result in a poor prognosis for IMVM fetuses is now a hot research topic.

The development of sulci and gyri is one of the most important markers of cortical maturation and is used as an indicator of cortical development [6]. Abnormal fetal cortical development is a major cause of dyskinesia, dysgnosia and behavioral disorders. An subjective ultrasonographic scoring system assessing fetal cortical development was developed by L. R. Pistorius to describe prenatal cortical folding that ranges from grade 0 (no development) to grade 5 (maximum development) based on the shape of the sulci and gyri [7]. Several studies have evaluated the cortical development of IMVM fetuses using this method and reported underdeveloped cortical structures [8, 9]. However, in these studies, only 2–3 ultrasound scans were performed, with no consistent systematic assessment. Moreover, these studies did not evaluate the prognosis of IMVM fetuses. In our clinical work, we have found that underdeveloped cortical structures and asymmetric cortical maturation are not rare in IMVM fetuses. If these features are an indicator of a poor prognosis and abnormal neurodevelopment, they contradict the incidence of abnormal or delayed neurodevelopment in infants with IMVM, which is approximately 7.9%, as mentioned above. A prospective cohort study was designed in which we performed sequential detailed assessments of cortical development in IMVM fetuses using ultrasound and examined whether asymmetrical cortical development occurred to address this issue. Moreover, we aimed to predict the prognosis of IMVM fetuses and the correlation between cortical development and infant outcomes.

## Methods

### Subjects

**Fig. 1:**
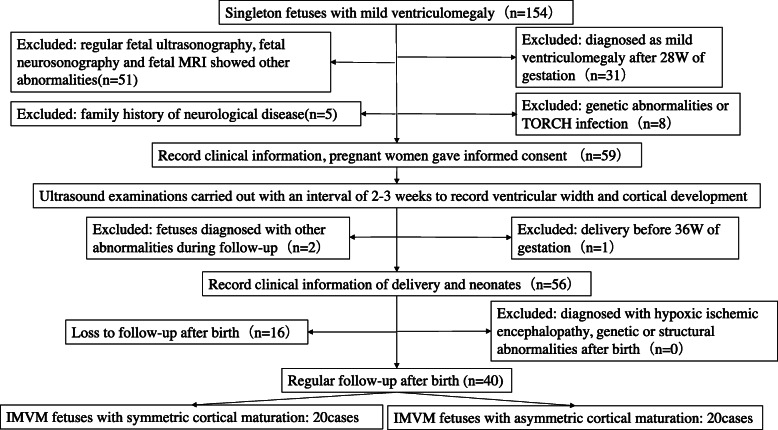
Participant flow chart

The present study was a prospective observational study involving singleton pregnant women who visited Peking University First Hospital from January 2016 to December 2019. The study was conducted in accordance with the Declaration of Helsinki. Our study was approved by the Peking University First Hospital Human Research Ethics Committee, and informed consent was obtained from all participants. The inclusion and exclusion criteria are shown in Fig. 1. We included fetuses between 21 weeks and 28 weeks of gestational age (GA) for two reasons. First, pregnant women undergo a second trimester ultrasound scan between 21 weeks and 23 weeks in our hospital; therefore, a fetus could be first diagnosed with mild ventriculomegaly at 21 weeks. Second, based on a previous study [7] and our experience, some sulci and gyri, such as the calcarine sulcus, might develop to grade 4 or 5 (maximum development) after 28 weeks, and thus an assessment of the cortical development process in fetuses who are diagnosed with IMVM after 28 weeks is of little value. We included 40 singleton pregnant women. The clinical information of each participant, including name, age, reproductive history and family history of nervous system disease, was recorded after inclusion. The gestational (postmenstrual) age was determined as the time from the first day of the last menstruation and was confirmed by a crown–rump length (CRL) measurement in the first trimester.

### Ultrasound

Transabdominal and transvaginal ultrasound examinations were conducted at an interval of 2–3 weeks using a Voluson E8 or Voluson E10 ultrasound device (GE Healthcare Ultrasound, Milwaukee, WI, USA) after the initial detection of ventriculomegaly. Regular ultrasound, including biometric measurements, was carried out, followed by neurosonography. Neurosonography was performed with a multiplanar assessment according to the International Society of Ultrasound in Obstetrics and Gynecology guidelines (ISUOG 2007) [10] Transabdominal neurosonography was performed with 3–5-MHz probes. Transvaginal neurosonography was performed with 5–9-MHz probes.

As a general neurosonographic assessment, the distal lateral ventricle atrial width was obtained in the transventricular plane, and the proximal lateral ventricle was assessed in coronal planes [10–12]. Progression of ventriculomegaly was defined as ventricular enlargement greater than 2 mm compared with the initial measurement. Stable ventriculomegaly was defined as an enlargement of less than 2 mm, and regressive ventriculomegaly was defined as a decrease of 2 mm or more or as a ventricular diameter of less than 10 mm [8, 13].

We also assessed the maturation of the parieto-occipital, calcarine and cingulate sulci of both hemispheres in IMVM fetuses at specific planes using the scoring method described by L. R. Pistorius [7]. The sulci we chose to examine were close to the midline, which would not be shaded by the fetal skull and enabled their continuous assessment throughout pregnancy using transabdominal and transvaginal neurosonography. The parieto-occipital sulcus was evaluated in the axial cephalic plane, the calcarine sulcus was evaluated in the coronal transcerebellar plane, and the cingulate sulcus was evaluated in the coronal transcaudate and transthalamic planes. A subjective score ranging from grade 0 to grade 5 was used to assess cortical development, where 0 is no development and 5 is the highest grade of maturation. More specifically, a grade 0 sulcus is not visible, a grade 1 sulcus displays a shallow indentation or echogenic dot shape, a grade 2 sulcus exhibits a broad V (width ≥ depth) shape, a grade 3 sulcus has a Y or narrow V (depth > width) shape, a grade 4 sulcus has an I- or J-shape, and a grade 5 sulcus shows branches from the grade 4 shape. Asymmetric sulcal maturation in the two hemispheres was defined as a difference of at least one grade between any sulci in both hemispheres. Furthermore, the fetuses were divided into the symmetric maturation group and the asymmetric maturation group.

### Magnetic resonance imaging (MRI)

All participants in this study underwent fetal MRI. Fetal MRI was performed using a 1.5-T MRI System (Multiva 1.5 T, Philips Healthcare, Best, The Netherlands) with a 32-channel cardiac-phased array surface placed over the gravid uterus for signal reception. The fetal head was scanned at the scanning center in the coronal, transverse, and sagittal planes using Half-Fourier acquisition single-shot turbo spin-echo (HASTE) sequences (repetition time of 1500 ms, echo time of 107–160 ms, slice thickness of 3 mm, slice gap of 0 mm, matrix of 280 × 205). Additionally, the fetal head was scanned in the transverse plane using T1 fast field echo (T1-FFE) sequences (repetition time of 15 ms, echo time of 7.5 ms, slice thickness of 3 mm, slice gap of 0 mm, matrix of 160 × 151) and diffusion weighted imaging (DWI) sequences (repetition time of 3249 ms, echo time of 90 ms, slice thickness of 3 mm, slice gap of 0 mm, matrix of 92 × 72). A 24 cm to 30 cm field of view (FOV) was used.

### Genetic and TORCH examinations

Genetic examinations of all fetuses were performed to exclude common genetic abnormalities, including a karyotype analysis, array-based comparative genome hybridization (aCGH) and single nucleotide polymorphism (SNP) array analysis, referring to the Database of Genomic Variants, Decipher Database, Online Mendelian Inheritance in Man (OMIM) database, PubMed, and other databases. Additionally, a TORCH (toxoplasmosis, other, rubella, cytomegalovirus and herpes simplex virus) examination was performed in all fetuses to exclude common infections.

### Prognostic analysis

Data on pregnancy outcomes (gender, Apgar score and perinatal and neonatal morbidity and mortality) were recorded for all cases. Pediatricians from the Department of Pediatrics in our hospital with professional training evaluated the neonatal behavioral neurological assessment (NBNA) scores for each included infant, including the general condition, action behavior, muscular tension, and primitive reflex, within 7 days of birth. Each parameter was assigned 0–2 points, with a total score of 40 and a score of greater than 36 defined as normal. The peripheral environment was kept quiet, and the temperature was maintained at a suitable level. The room temperature was maintained at approximately 26 °C. All evaluation parameters were assessed in sleeping children 1 h after being fed.

Cranial ultrasound examinations of infants were performed by an experienced sonographer using an ATL 5000 Unit (Philips, Best, The Netherlands) within 6 months after birth. Abnormalities of the neonatal brain were recorded.

At 6, 12 and 18 months after birth, all included infants were assessed using the Ages and Stages Questionnaire, Third Edition (ASQ-3). Parents answered 30 questions covering 5 domains of development, including communication, gross motor, fine motor, problem-solving, and adaptive skills. Parents were instructed to try activities with their child to facilitate an accurate assessment. A pass/fail score was assigned for each area of development. The presence of a problem in any domain screened, defined as 2 standard deviations below the mean area score, was considered to indicate delayed development. Moreover, some of the included children were assessed with Bayley Scales of Infant Development, First Edition (BSID-I) at 6, 12 and 18 months. Experienced physicians in our hospital used the BSID-I to assess neurodevelopment, including the mental developmental index (MDI) and psychomotor developmental index (PDI). Scores of less than 79 on the MDI or PDI were considered to indicate a low level of development. Other infants were unable to undergo this assessment due to the COVID-19 outbreak or because they lived far from our hospital.

### Statistical analysis

Data were analyzed using SPSS version 21 for Mac (SPSS Inc., Chicago, IL). Categorical variables are reported as percentages and continuous variables are presented as means or median values. Student’s t-test for independent samples and Pearson’s chi-square tests were used to compare quantitative and qualitative data between the symmetric maturation and asymmetric maturation groups.

## Results

### Subjects

Forty fetuses were included in this study: 20 (50.0%) of them showed symmetric cortical maturation and the other 20 (50.0%) exhibited asymmetric maturation. The demographic characteristics of the two groups are presented in Table 1. The mean maternal age of the symmetric maturation group was 32.20 years (range 27–38 years), the mean gestational age at birth was 38.95 weeks (range 37–41 weeks), and the mean birthweight was 3574.00 g (range 3005–4190 g). The mean maternal age of the asymmetric maturation group was 31.10 years (range 27–37 years), the mean gestational age at birth was 38.56 weeks (range 37–40 weeks), and the mean birthweight was 3464.94 g (range 2950–3835 g). Apgar scores at 1 and 5 min were 10 in all cases. Statistically significant differences in the aforementioned parameters were not observed between the symmetric and asymmetric maturation groups. (*p* values were 0.29, 0.30, 0.29, 1.00 and 1.00, respectively).

**Table 1:** Demographic characteristics

	Symmetric maturation group (*n* = 20)	Asymmetric maturation group(*n* = 20)	*P* value
Maternal age	32.20 ± 3.46	31.10 ± 2.94	0.29
Primipara			
Yes	11 (55.0%)	12 (66.7%)	0.52
No	9 (45.0%)	8 (33.8%)	
Fetus gender			
Male	11 (55.00%)	13 (65.00%)	0.75
Female	9 (45.00%)	7 (35.00%)	
GA at delivery	38.95 ± 1.23	38.56 ± 1.04	0.30
Birth weight (g)	3574.00 ± 342.25	3464.94 ± 284.68	0.29
Apgar score			
1 min	10 ± 0	10 ± 0	1.00
5 min	10 ± 0	10 ± 0	1.00

Data are given as mean ± standard deviation or number (percentage). *GA* Gestational age (weeks)

### Neurodevelopment

**Fig. 2:**
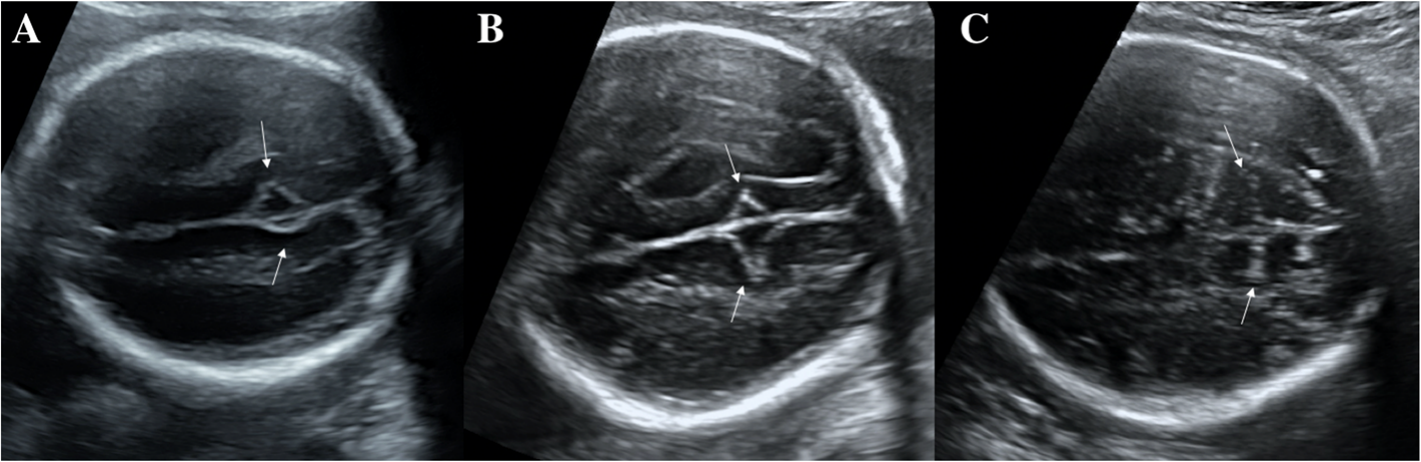
Asymmetry in the development and ‘catch-up growth’ of parieto-occipital sulcus in a IMVM fetus with unilateral ventriculomegaly. Ultrasound image of parieto-occipital sulcus on axial cephalic plane at 25 weeks (**a**), 28 weeks(**b**) and 31 weeks (**c**) of gestation. **a** The parieto-occipital sulcus was grade 1 on one side and was grade 2 on the other side. **b** The parieto-occipital sulcus was grade 2 on one side and was grade 3 on the other side. **c** The parieto-occipital sulcus was grade 4 on both sides

**Fig. 3:**
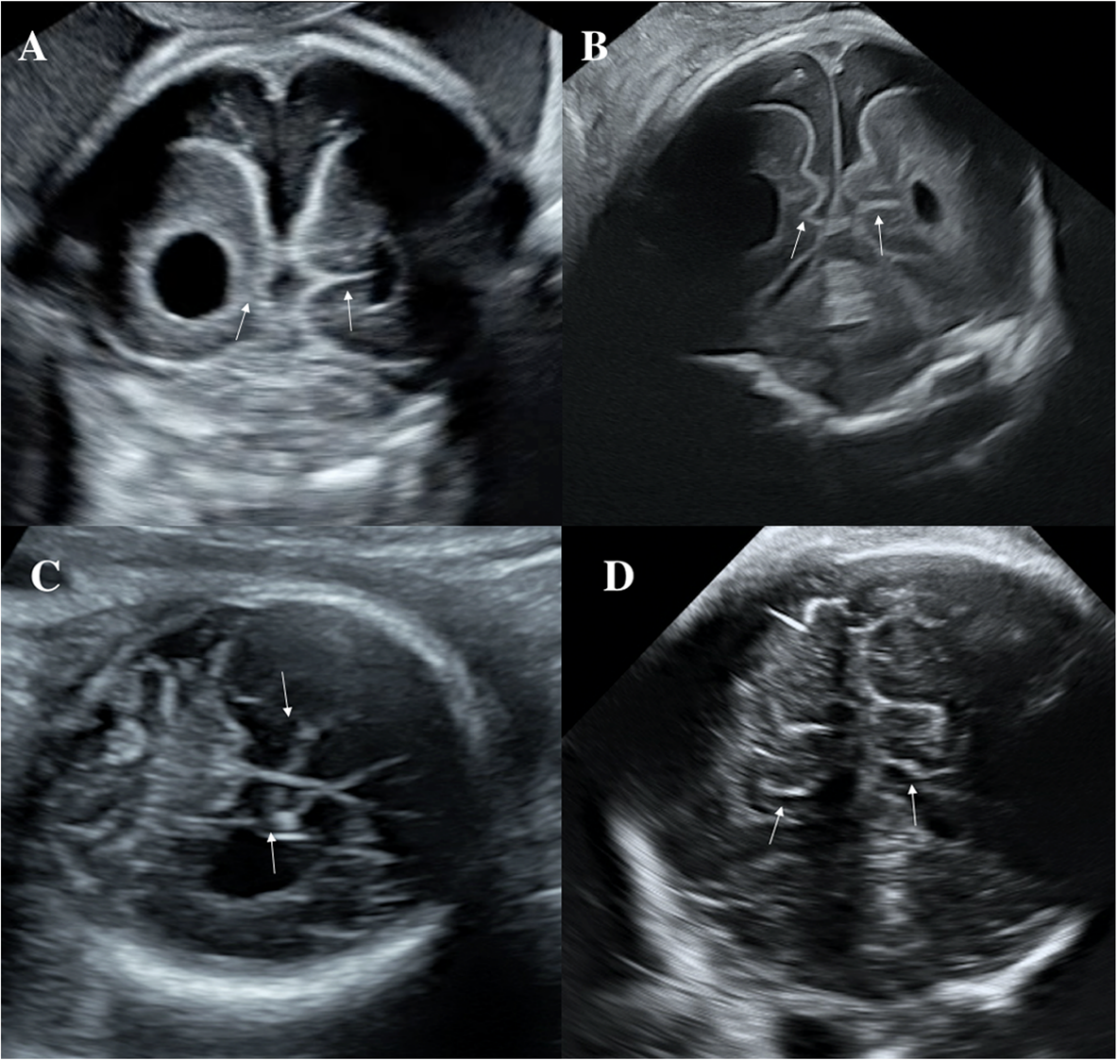
Asymmetry in the development and ‘catch-up growth’ of calcarine sulcus in a IMVM fetus with unilateral ventriculomegaly. Ultrasound image of calcarine sulcus on coronal transcerebellar plane at 25 weeks (**a**), 27 weeks (**b**), 30 weeks (**c**) and 32 weeks (**d**) of gestation. **a** the calcarine sulcus was grade 1 on one side and was grade 2 on the other side. **b** the calcarine sulcus was grade 2 on one side and was grade 4 on the other side. **c** the calcarine sulcus was grade 4 on both sides. **d** the calcarine sulcus was grade 5 on both sides

**Fig. 4:**
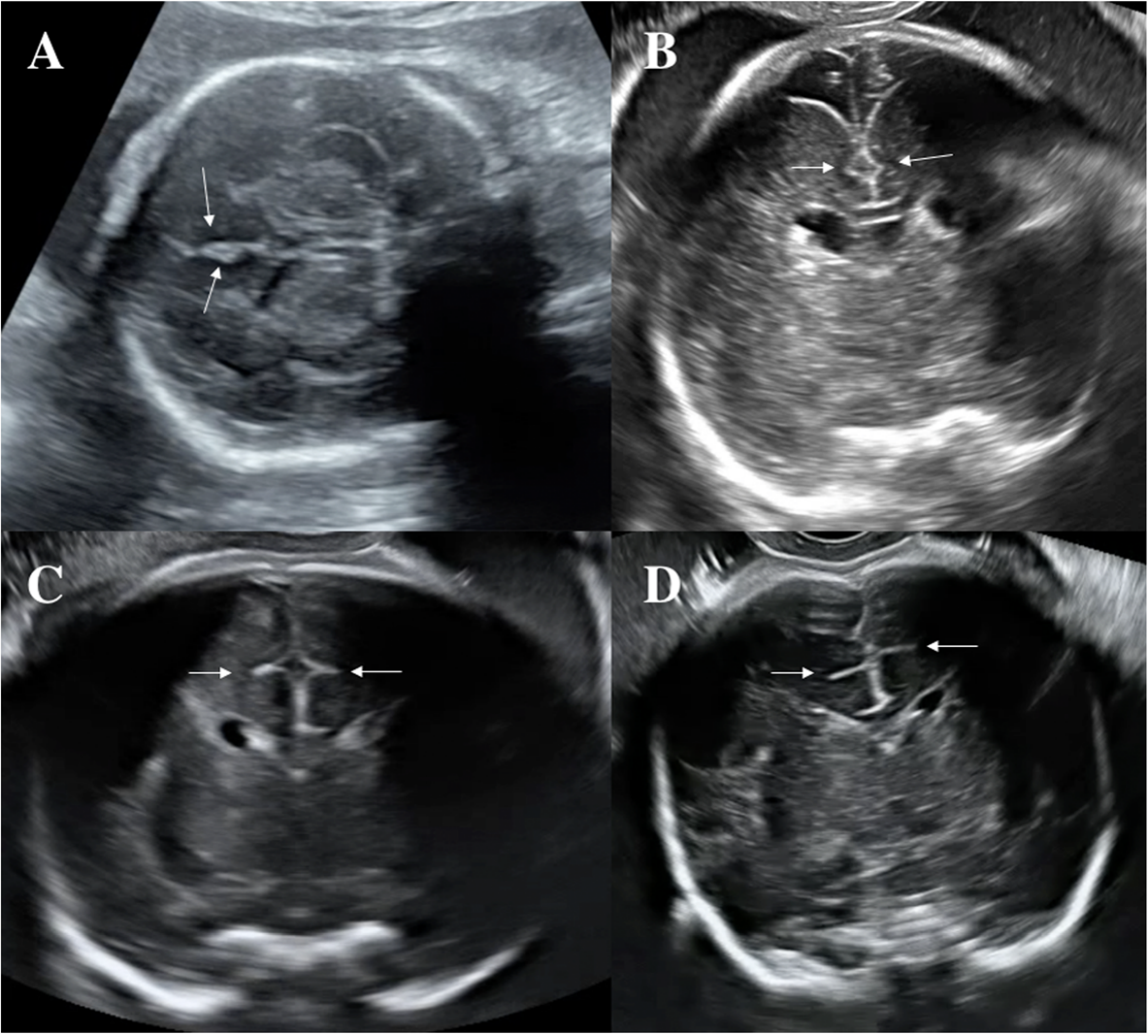
Asymmetry in the development and ‘catch-up growth’ of cingulate sulcus in a IMVM fetus with unilateral ventriculomegaly. Ultrasound image of cingulate sulcus on coronal transcaudate and transthalamic plane at 27 weeks (**a**), 30 weeks (**b**), 32 weeks (**c**) and 34 weeks (**d**) of gestation. **a** The cingulate sulcus was grade 0 on one side and was grade 1 on the other side. **b** The cingulate sulcus was grade 2 on one side and was grade 3 on the other side. **c** The cingulate sulcus was grade 3 on both sides. **d** The calcarine sulcus was grade 4 on both sides

**Fig. 5:**
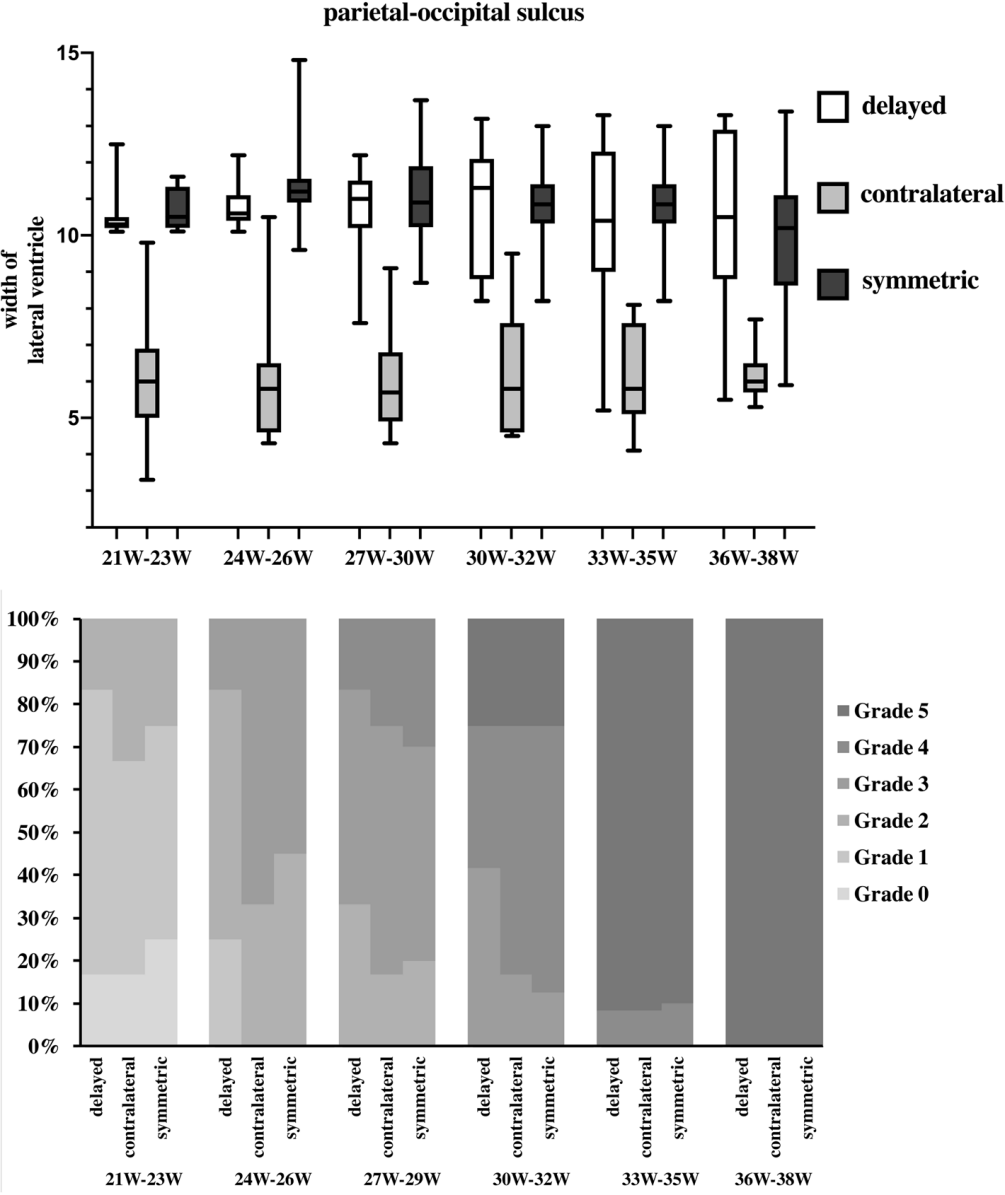
The width of lateral ventricle and cortical grading of IMVM fetuses with parietal-occipital sulcus asymmetry and IMVM fetuses with symmetric cortical development at different gestational ages. The upper part of this figure are box plots that describe the change in width of lateral ventricle. The box plots show minimum, median and maximum values and the 1st and 3rd quartiles. The lower part of the figure are percentage bar charts that describe the distribution of cortical grading scores. Delayed: the relatively delayed side of IMVM fetuses presenting parietal-occipital sulcus asymmetry. Contralateral: the contralateral side of IMVM fetuses presenting parietal-occipital sulcus asymmetry. Symmetric: IMVM fetuses presenting symmetric cortical development

**Fig. 6:**
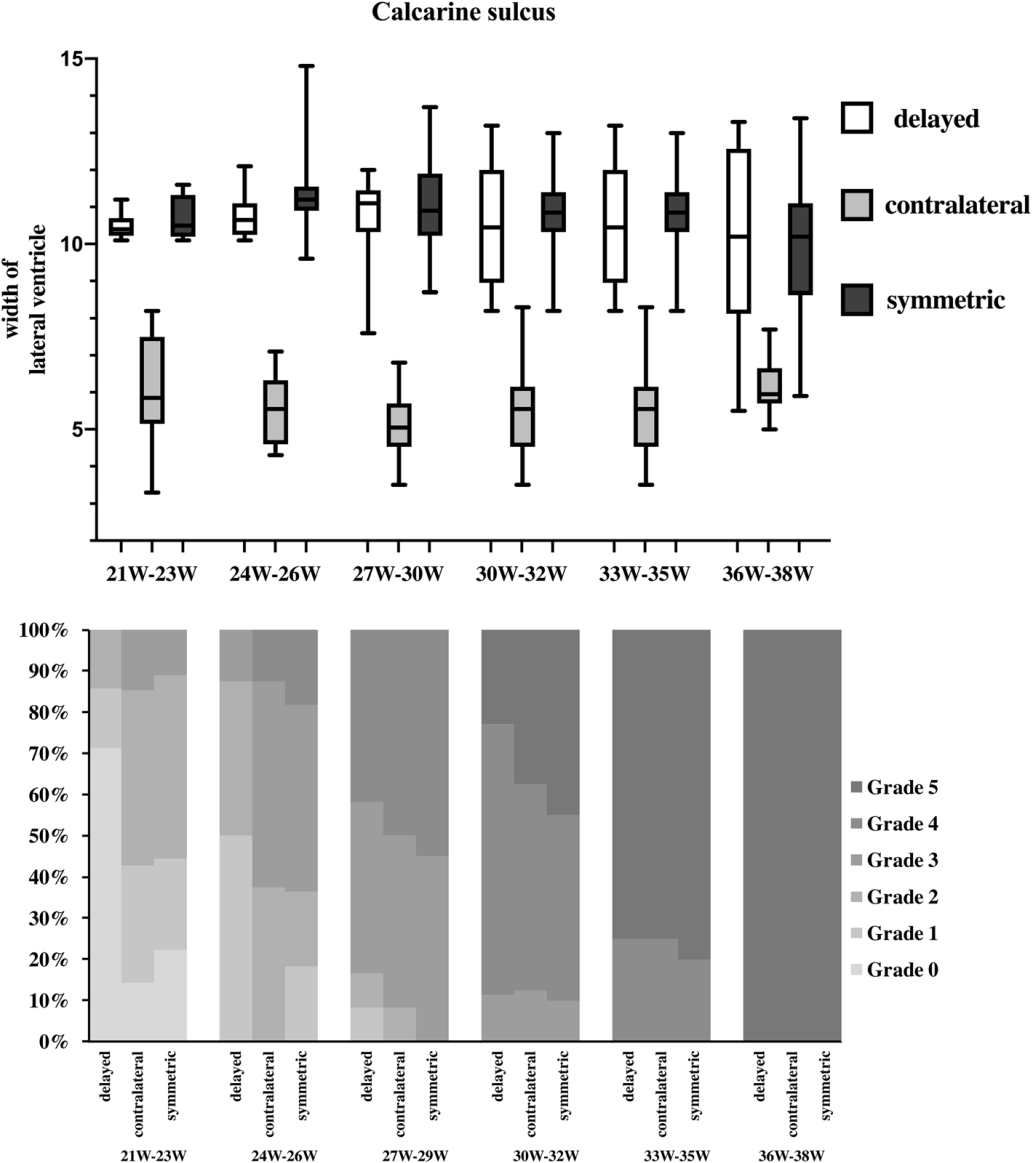
The width of lateral ventricle and cortical grading of IMVM fetuses with calcarine sulcus asymmetry and IMVM fetuses with symmetric cortical development at different gestational ages. The upper part of this figure are box plots that describe the change in width of lateral ventricle. The box plots show minimum, median and maximum values and the 1st and 3rd quartiles. The lower part of the figure are percentage bar charts that describe the distribution of cortical grading scores. Delayed: the relatively delayed side of IMVM fetuses presenting calcarine sulcus asymmetry. Contralateral: the contralateral side of IMVM fetuses presenting calcarine sulcus asymmetry. Symmetric: IMVM fetuses presenting symmetric cortical development

**Fig. 7:**
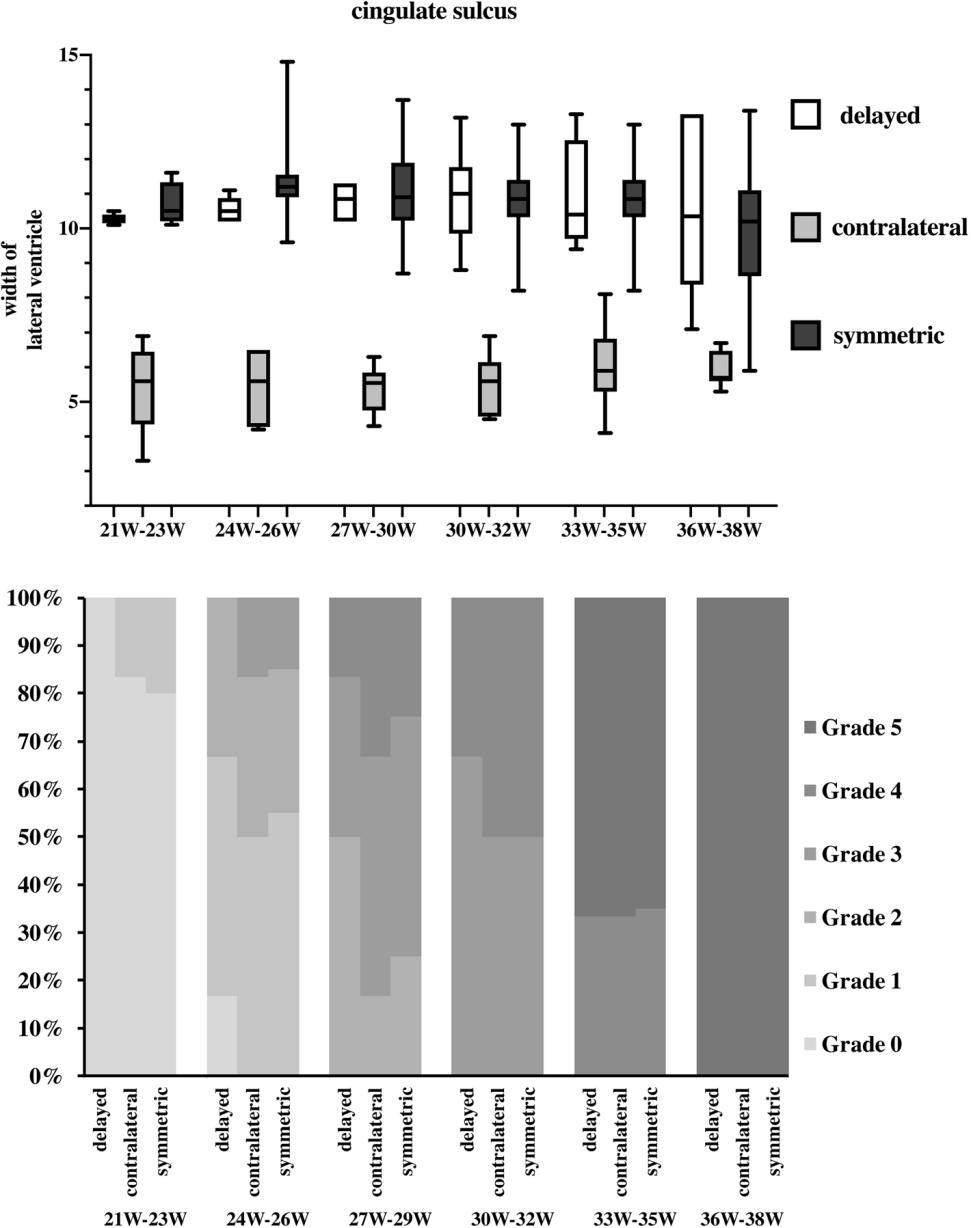
The width of lateral ventricle and cortical grading of IMVM fetuses with cingulate sulcus asymmetry and IMVM fetuses with symmetric cortical development at different gestational ages. The upper part of this figure are box plots that describe the change in width of lateral ventricle. The box plots show minimum, median and maximum values and the 1st and 3rd quartiles. The lower part of the figure are percentage bar charts that describe the distribution of cortical grading scores. Delayed: the relatively delayed side of IMVM fetuses presenting cingulate sulcus asymmetry. Contralateral: the contralateral side of IMVM fetuses presenting cingulate sulcus asymmetry. Symmetric: IMVM fetuses presenting symmetric cortical development

The mean GA at the first diagnosis of mild ventriculomegaly was 23.50 weeks (range 21–28 weeks) in the symmetric maturation group and 24.00 weeks (range 22–27 weeks) in the asymmetric maturation group. In the symmetric maturation group, 15 (75.0%) fetuses presented bilateral ventriculomegaly, and 5 (25.0%) fetuses presented unilateral ventriculomegaly. In the asymmetric maturation group, 19 (95.0%) fetuses presented unilateral ventriculomegaly, and 1 (5.0%) fetus presented bilateral ventriculomegaly. The incidence of unilateral ventriculomegaly was significantly different between the symmetric and asymmetric maturation groups (*p* = 0.00). The prenatal assessment of the lateral ventricle of IMVM fetuses is presented in Table 2. Notably, all developmental delays occurred in the hemisphere with ventriculomegaly compared with the other side in fetuses presenting unilateral ventriculomegaly. Only one fetus in the asymmetric group presented bilateral ventriculomegaly. In this fetus, the right ventriculomegaly was regressive, and the lateral width returned to normal at 32 weeks. The lateral width of the left side was stable throughout pregnancy. As expected, relatively delayed development occurred on the stable side. The sulcal development of the asymmetric maturation group is presented in Table 3. Eight (40.0%) fetuses showed asymmetric development of one sulcus, 10 (50.0%) fetuses showed asymmetric development of two sulci, 2 (10.0%) fetuses showed asymmetric development of three sulci, and 34 sulci with relatively delayed development were observed compared with sulci in the other hemisphere. The mean GA at the first diagnosis of relatively delayed development was 24.23 weeks (range 22–27 weeks) for the parieto-occipital sulcus, 24.71 weeks (range 22–28 weeks) for the calcarine sulcus, and 26.43 weeks (range 25–29 weeks) for the cingulate sulcus. According to our assessment, all sulci with delayed development underwent ‘catch-up growth’ and developed to the same grade as the sulci in the other hemisphere. The mean GA at which the two sides developed to the same grade was 29.40 weeks (range 24–32 weeks) for the parieto-occipital sulcus, 29.30 weeks (range 24–31 weeks) for the calcarine sulcus and 31.27 weeks (range 27–34 weeks) for the cingulate sulcus. Figures 2, 3 and 4 show the ‘catch-up growth’ patterns of the parieto-occipital sulcus, calcarine sulcus and calcarine sulcus, respectively. The relatively delayed sulci showed one or two grades of delay compared with the other side, but none showed three or more grades of delay. The change in the width of the lateral ventricle and development of the included sulci are shown in Figs. 5, 6 and 7 at two-week intervals during pregnancy. For statistical analyses, we used the width of the more dilated side in fetuses with bilateral ventriculomegaly. As shown in Figs. 5, 6 and 7, the parieto-occipital, calcarine and cingulate sulci of all IMVM fetuses presenting symmetric or asymmetric cortical maturation in our study developed to grade 5 before birth. Figures 5, 6 and 7 and Table 2 also show a regression of ventriculomegaly in only some fetuses with IMVM, while ventriculomegaly remained stable or even progressed in other fetuses during the course of cortical maturation. The median value of the lateral ventricle remained stable in both the asymmetric and symmetric groups. The change in the width of the lateral ventricle was not significantly different between the two groups (*p* = 0.51).

**Table 2:** Prenatal assessment of lateral ventricle of IMVM fetuses

	Symmetric maturation group (*N* = 20)	Asymmetric maturation group (*N* = 20)	*P* value
GA of IMVM first diagnosed	23.50 ± 2.07	24.00 ± 2.16	0.47
Lateral ventriculomegaly			
Unilateral	5 (25.0%)	19 (95.0%)	0.00
Bilateral	15 (75.0%)	1 (5.0%)	
Width change between first diagnosed			
US and last US before born^a^:	*N* = 35	*N* = 21	0.51
Progressive:	10 (28.6%)	3 (14.3%)	
Stable:	11 (31.4%)	8 (38.1%)	
Regressive:	14 (40.0%)	9 (42.9%)	

Data are given as mean ± standard deviation or number (percentage). *GA* Gestational age (weeks). US Ultrasonography. ^a^ In lateral ventricle with ventriculomegaly

**Table 3:** Prenatal cortical maturation of asymmetric maturation group

	*N* = 20
Number of relatively retarded sulci in a fetus	
1	8 (40.0%)
2	10 (50.0%)
3	2 (10.0%)
Category of relatively retarded sulci	
Parieto-occipital sulcus	12 (60.0%)
Calcarine sulcus	16 (80.0%)
Cingulate sulcus	6 (30.0%)
GA of first diagnosed with relatively retardation	
Parieto-occipital sulcus	24.23 ± 1.40
Calcarine sulcus	24.71 ± 1.60
Cingulate sulcus	26.43 ± 0.89
GA of bilateral sulci grew into symmetry	
Parieto-occipital sulcus	29.40 ± 1.71
Calcarine sulcus	29.30 ± 2.29
Cingulate sulcus	31.27 ± 1.64

Data are given as mean ± standard deviation or number (percentage). *GA* Gestational age (weeks)

### Fetal MRI

All of the fetuses showed no central nervous system abnormalities except mild ventriculomegaly. The mean GA of fetal MRI examination was 27.30 weeks and the mean width of lateral ventricle presenting ventriculomegaly was 11.2 mm in symmetric maturation group. The mean gestational age of fetal MRI examination was 28.20 weeks and the mean width of lateral ventricle presenting ventriculomegaly was 11.7 mm in asymmetric maturation group. The aforementioned parameters showed no statistically significant differences between the symmetric and asymmetric maturation groups (*P* values are 0.43 and 0.67 respectively).

### Prognoses

The NBNA scores of all included infants were greater than 36 within the seventh day after birth. The cranial ultrasounds of all infants showed no abnormalities other than mild ventriculomegaly.

The follow-up results are shown in Table 4. At the end of our study, the included children were of different ages. In the symmetric group, 6 children were between 6 and 12 months old, 3 children were between 12 and 18 months old, and 11 children were over 18 months old. In the asymmetric group, 7 children were between 6 and 12 months old, 7 children were between 12 and 18 months old, and 6 children were over 18 months old. We assessed the neurodevelopment of all the included children at 6, 12 and 18 months of age using the ASQ-3. Some of the included children were also assessed using the BSID-I at 6, 12 and 18 months of age. Scores in the normal range were recorded for all included infants, including those who underwent the ASQ-3 assessment and both the ASQ-3 and BSID-I assessments. None of the included children were defined as having delayed development according to the results of the assessments at 6, 12 or 18 months of age, and no statistically significant differences in the results were observed between the children in the two groups. Furthermore, at the end of our study, none of the included children showed neurological diseases other than mild fetal ventriculomegaly.

**Table 4:** Prognosis of IMVM fetuses after birth

	Cases underwent ASQ-3	Cases underwent ASQ-3 with normal result	*P* value	Cases underwent BSID-I	Result of Bayley-I			
					PDI (mean ± SD)	*P* Value	MDI (mean ± SD)	*P* Value
6 months								
SMG	20	20	1.00	15	107.27 ± 12.44	0.57	107.87 ± 16.99	0.79
AMG	20	20		10	110.50 ± 13.49		109.70 ± 14.98	
12 months								
SMG	14	14	1.00	7	106.86 ± 12.21	0.56	106.62 ± 12.52	0.64
AMG	13	13		3	113.00 ± 15.12		103.67 ± 9.03	
18 months								
SMG	11	11	1.00	2	93.00 ± 7.00	/	102.50 ± 2.50	/
AMG	6	6		0	/		/	

*SMG* IMVM fetuses in symmetric maturation group, *AMG* IMVM fetuses in asymmetric maturation group

## Discussion

In this study, we observed the maturation process of selected sulci after 21 weeks of gestation and the prognosis of 40 infants with IMVM. All fetuses with relatively delayed development in one hemisphere compared with the other hemisphere occurred in the hemisphere with ventriculomegaly or with continuously stable ventriculomegaly and not in the hemisphere with a normal lateral ventricle or with regressive ventriculomegaly. Therefore, we infer that asymmetric cortical maturation is not random and tends to occur in infants with IMVM presenting unilateral ventriculomegaly or asymmetric bilateral ventriculomegaly.

Several studies have reported cortical development in IMVM fetuses using the same scoring method as that used in the present study [8, 9]. To our knowledge, this study is the first to report the ‘catch-up growth’ pattern in IMVM fetuses presenting asymmetric cortical maturation. In our study, the relative delay in the development of calcarine and parieto-occipital sulci occurred at approximately 24 weeks and relative delay in the development of the cingulate sulcus occurred at approximately 26 weeks. The relatively delayed sulci underwent a process of ‘catch-up growth’ in which the sulci of the two hemispheres ultimately grew symmetrically and developed to the maximum grade before birth.

Several studies have reported asymmetrical cortical development in ‘normal fetuses’. Hering-Hanit [14] and Kivilevitch [15] reported left-right hemisphere asymmetry by measuring the diameters of both hemispheres. Pistorius [7] evaluated fetal cortical development using ultrasound and reported asymmetry in at least one examination in 20 of the 28 fetuses. Notably, these studies did not follow the fetuses to exclude those who were diagnosed with genetic or structural abnormalities after birth. Some of these studies did not carry out fetal MRI, since fetal MRI is vital for identifying additional central nervous system anomalies [16, 17]. As a result, further investigation is needed to assess the symmetry of cortical development in normal fetuses and compare the cortical growth patterns between normal fetuses and IMVM fetuses.

As mentioned above, all the cases of relatively delayed development in one hemisphere compared with the other hemisphere occurred in the hemisphere with ventriculomegaly or with continuously stable ventriculomegaly. The relatively delayed sulci underwent a process of ‘catch-up growth’ and the sulci of the two hemispheres ultimately grew symmetrically. However, only 42.9% of the enlarged lateral ventricles in IMVM fetuses presenting asymmetric cortical maturation showed regressive ventriculomegaly before birth, and 57.1% of the enlarged lateral ventricles showed stable or progressive ventriculomegaly. Therefore, we infer that delayed cortical development is not the reason for ventriculomegaly, and the possible correlations between ventriculomegaly and cortical development and between cortical maturation and width changes associated with ventriculomegaly remain unclear.

All the included infants underwent a cranial ultrasound examination after birth, and none were diagnosed with abnormalities other than mild ventriculomegaly, which suggests that prenatal neurosonography resulted in no false-negative diagnoses. Regarding the prognostic estimation, all infants underwent the NBNA 7 days after birth and ASQ-3 at 6, 12 and 18 months. Additionally, some of the included infants underwent both the ASQ-3 and BSID-I. The result of these tests showed normal neurodevelopment in all cases. Many investigators consider the Bayley scales the gold standard for assessing infant development. However, its universal clinical applicability is limited due to its high cost, timing, and requirement for administration by trained professionals [18]. Some of our participants were unable to undergo BSID-I due to the COVID-19 outbreak or because they lived far from our hospital, but all participants underwent the ASQ-3. Multiple studies support ASQ’s easy administration, short completion time, ease of interpretation, and capacity to identify children who have suspected developmental delays [19–21]. According to a systematic review, the sensitivity and specificity of the ASQ-3 are reliable compared with the Bayley scales [22]. Therefore, the results of the neurodevelopment assessment using the ASQ-3 in our study are reliable.

Fetuses in the asymmetric maturation group showed no statistically significant difference in neurodevelopment within 18 months after birth compared with the symmetric maturation group. Therefore, such mild asymmetric cortical maturation may be a physiological phenomenon. However, close observation of cortical maturation and whether the sulci undergo ‘catch-up growth’ during pregnancy is essential for IMVM fetuses with asymmetric cortical maturation. Moreover, only 6 children in the asymmetric group were over 18 months of age at the end of the study, further analyses with more children are needed to prove this conclusion.

According to a recent meta-analysis, the rate of abnormal or delayed neurodevelopment in IMVM infancy is approximately 7.9% [5]. In our study, none of the participants showed abnormal or delayed neurodevelopment. One possible explanation is that the fetuses in our study underwent thorough prenatal examinations, with the strict exclusion of participants using neurosonography. We ensured that all fetuses with other structural abnormalities were excluded and that no single false-negative prenatal diagnosis occurred in our study. Another possible explanation is that we followed the participants up to 18 months; however, neurodevelopmental disorders reported in previous research, including autism spectrum disorder and epilepsy [23], may require a longer follow-up time for diagnosis. Moreover, the sample size of our study was too small to illustrate the incidence of neurodevelopmental disorders in IMVM fetuses presenting asymmetric cortical maturation, which is also a limitation of our study. Further research with larger sample sizes is needed to evaluate the long-term prognosis of IMVM fetuses presenting asymmetric cortical development.

## Conclusions

IMVM fetuses may show mild asymmetric cortical maturation in the second trimester, but the relatively delayed sulci undergo ‘catch-up growth’. Limited cases have shown that the neurodevelopment of IMVM fetuses presenting asymmetric cortical maturation and ‘catch-up growth’ is not statistically significantly different from IMVM fetuses presenting symmetric cortical maturation. This ‘catch-up growth’ pattern may be a physiological phenomenon.

## Data Availability

The datasets collected during the current study are available from the corresponding author upon reasonable request.

## Abbreviations

IMVM: Isolated mild ventriculomegaly
GA: Gestational age
NBNA: Neonatal behavioral neurological assessment
ASQ-3: Ages and Stages Questionnaire, Third Edition
BSID-I: Bayley Scales of Infant Development, First Edition
